# Estimating KIR Haplotype Frequencies on a Cohort of 10,000 Individuals: A Comprehensive Study on Population Variations, Typing Resolutions, and Reference Haplotypes

**DOI:** 10.1371/journal.pone.0163973

**Published:** 2016-10-10

**Authors:** Cynthia Vierra-Green, David Roe, Jyothi Jayaraman, John Trowsdale, James Traherne, Rui Kuang, Stephen Spellman, Martin Maiers

**Affiliations:** 1 Center for International Blood and Marrow Transplant Research, Minneapolis, Minnesota, United States of America; 2 Bioinformatics Research, National Marrow Donor Program, Minneapolis, Minnesota, United States of America; 3 Department of Pathology, University of Cambridge, Cambridge, United Kingdom; 4 Department of Computer Science and Engineering, University of Minnesota, Minneapolis, Minnesota, United States of America; Ohio State University Wexner Medical Center, UNITED STATES

## Abstract

The killer cell immunoglobulin-like receptors (KIR) mediate human natural killer (NK) cell cytotoxicity via activating or inhibiting signals. Although informative and functional haplotype patterns have been reported, most genotyping has been performed at resolutions that are structurally ambiguous. In order to leverage structural information given low-resolution genotypes, we performed experiments to quantify the effects of population variations, reference haplotypes, and genotyping resolutions on population-level haplotype frequency estimations as well as predictions of individual haplotypes. We genotyped 10,157 unrelated individuals in 5 populations (518 African American[AFA], 258 Asian or Pacific Islander[API], 8,245 European[EUR], 1,073 Hispanic[HIS], and 63 Native American[NAM]) for KIR gene presence/absence (PA), and additionally half of the AFA samples for KIR gene copy number variation (CNV). A custom EM algorithm was used to estimate haplotype frequencies for each population by interpretation in the context of three sets of reference haplotypes. The algorithm also assigns each individual the haplotype pairs of maximum likelihood. Generally, our haplotype frequency estimates agree with similar previous publications to within <5% difference for all haplotypes. The exception is that estimates for NAM from the U.S. showed higher frequency association of cB02 with tA01 (+14%) instead of tB01 (-8.5%) compared to a previous study of NAM from south of the U.S. The higher-resolution CNV genotyping on the AFA samples allowed unambiguous haplotype-pair assignments for the majority of individuals, resulting in a 22% higher median typing resolution score (TRS), which measures likelihood of self-match in the context of population-specific haplo- and geno-types. The use of TRS to quantify reduced ambiguity with CNV data clearly revealed the few individuals with ambiguous genotypes as outliers. It is observed that typing resolution and reference haplotype set influence haplotype frequency estimates. For example, PA resolution may be used with reference haplotype sets up to the point where certain haplotypes are gene-content subsets of others. At that point, CNV must be used for all genes.

## Introduction

Human chromosome 19 contains a ~150–250 kilobase region that encodes 16 genes of the natural killer cell immunoglobulin-like receptor (KIR) family. These genes are ~10–15 kilobases long and evolved via tandem duplication and deletion during primate evolution[1][2]. These processes have varied gene content and copy number, such that dozens of haplotypes have already been reported[3][4] in Europeans alone. Each gene can be present between 0–4 times in any given haplotype. Thus, presence/absence genotypes differ from copy number genotypes, and the recessive status of genes can be hidden if only the presence is known. KIR receptors recognize Human Leukocyte Antigen (HLA) class I molecules and mediate Natural Killer (NK) cell cytotoxicity via activating or inhibiting signals. These receptor-ligand pairs coevolved under selection pressures from reproduction and pathogenic defense[5][6].

Selective pressures have divided KIR haplotypes into two roughly balanced groups, A and B. The canonical A haplotype contains seven genes and two pseudo genes; it is the most common single haplotype with a frequency of at least 50%, with an exception in East Asia[7]. B haplotypes vary widely in gene content and contain more activating receptors than group A haplotypes. B haplotypes are more structurally diverse, with all their genes exhibiting variable copy number, although very rare in *KIR3DL3* and *KIR3DL2*[4][8].

Informative and functional structural patterns have been reported via evolutionary and therapeutic-outcomes analysis. For example, Hilton et al. provided an evolutionary report on how specific subgroups of HLA-C ligands preferentially associate with certain KIR genes and how those genes are preferentially located on certain A or B haplotypes[9]. Another example is a clinical report from Cooley et al. on preferential outcomes for certain B haplotypes in unrelated transplantation for Acute Myelogenous Leukemia[10]. These studies suggest it is valuable to interpret KIR in its structural context and doing so may help resolve ambiguous or contradictory associations found across a wide variety of low-resolution studies.

Within this context, our collective knowledge of structural diversity at the gene level is still coarse, especially for unrelated and non-European populations. Only a couple-dozen fully sequenced haplotypes have been deposited in Genbank for the KIR region[11][1][3], although population haplotype frequencies have been reported in many families or CNV-based studies. There have been no unrelated population studies using completely cis-linked haplotypes. Most typings–and therefore studies–have been conducted at gene PA or less often at CNV resolution due to technical and economic considerations of sequencing technologies relative to the characteristics of this large and repetitive region. Haplotypic interpretation can be ambiguous under both PA and CNV resolutions, although the extent of improvement for CNV has not been widely reported.

In order to leverage structural information given low-resolution genotypes, we performed experiments to quantify the effects of population variations, reference haplotypes, and genotyping resolution on population-level haplotype frequency estimations as well as predictions of individual haplotypes.

We generated haplotype frequency estimates for over 10,000 individuals in five populations. We reported on the effects of reference haplotype set and resolution, and documented the inaccuracies that may occur when using low resolution and large sets of reference haplotypes. These analytical methods and novel predictions may benefit therapeutic research such as hematopoietic stem cell transplants.

## Materials and Methods

### Subjects and Populations

**Fig 1:**
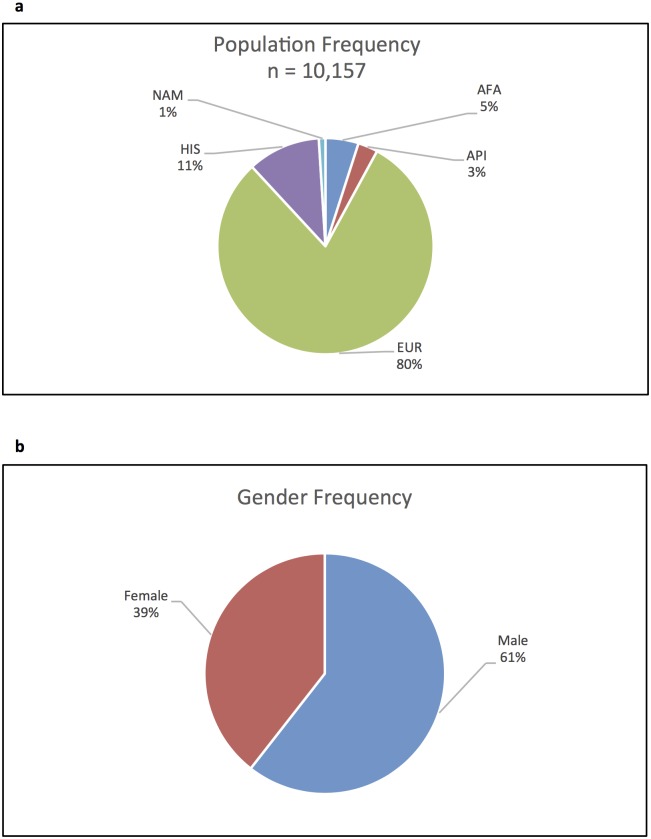
Population demographics. Cohort frequencies are depicted by population a) and gender b).

DNA samples for 4,131 recipients, 4,665 donors, and 1,361 umbilical cord units (10,157 total) from unrelated hematopoietic stem transplants were obtained from the Center for International Blood and Marrow Transplant Research (CIBMTR) Research Repository[12]. All subjects provided written informed consent for participation in research and the study and consent were approved by the National Marrow Donor Program Institutional Review Board. The cohort consisted of five self-described racial/ethnic populations: African American (AFA) 518 (5%), Asian Pacific Islander (API) 258 (3%), European (EUR) 8245 (81%), Hispanic (HIS) 1073 (11%), and Native American (NAM) 63 (1%) (Fig 1).

### KIR Genotyping

Genotyping was performed via sequence-specific primer-directed polymerase chain reaction (PCR) amplification (PCR-SSP) and sequence-specific oligonucleotide hybridization (PCR-SSO) for the PA of 16 genes: *KIR2DL1-5*, *KIR2DS1-5*, *KIR3DL1-3*, *KIR3DS1*, *KIR2DP1*, and *KIR3DP1*[13][14]. There were no missing data for the protein-coding genes, although 10% of *KIR2DP1*s, and 17% of *KIR3DP1*s were unavailable. Additionally, all AFAs for which sample was available (52%) were genotyped via quantitative PCR (qPCR) at CNV resolution for all genes[4].

### Reference haplotypes

**Fig 2:**
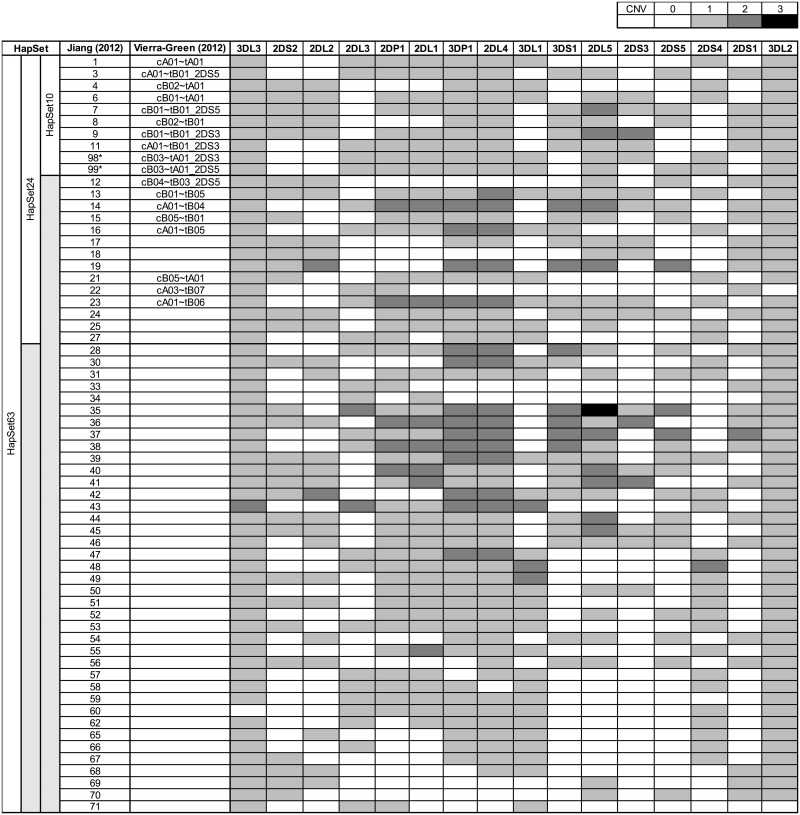
Reference haplotype sets. Each of 63 haplotypes depicted as copy number variants of 16 KIR genes. Copy number is labeled as shades of grey, where white boxes indicate 0 copies, and the darkest shade of grey indicates the highest variant number: 3. The haplotypes are primarily from individuals of European descent. a) HapSet10: 10 haplotypes from a study of 12 unrelated individuals. b) HapSet24: haplotypes with a frequency of > = 0.1% from Jiang et al. c) HapSet63: all haplotypes from Jiang et al. Haplotypes are labeled by number from the original publication and via Vierra-Green nomenclature when applicable. * cB03~tA01 is not in Jiang 2010; its *KIR2DS3* and *KIR2DS5* variants are arbitrarily labeled as 98 and 99. Note, haplotypes 2, 5 and 10 from Jiang (2012) represent alternative forms of gene content haplotypes based on allelic variation of *KIR2DS4* (1, 4 and 6 respectively) and are, therefore, not included in the table.

In Fig 2, haplotypes are labeled both by the number from the Jiang et al.[4] and the nomenclature from Vierra-Green et al.[15] when applicable. An example of the latter is ‘cA01~tB02’. ‘~’ = the large recombination hotspot between *KIR3DP1* and *KIR2DL4*. ‘c’ = centromeric (i.e. relatively proximal) region of the haplotype relative to the hotspot, ‘t’ = telomeric (i.e. relatively distal) region relative to the hotspot. ‘A’ = region from the A haplotype group, ‘B’ = region from the B haplotype group. ‘01’, ‘02’ = a unique label for the region within the A and B groups. Therefore ‘cA01~tB02’ is a full-length haplotype comprised of the first centromeric A region in *cis* with the second telomeric B region. Numeric haplotype labels are occasionally combined, as in ‘49/51’. This indicates CNV duplication/ambiguity under PA conditions; i.e., ‘49’ and ‘51’ are distinct haplotypes for CNV resolution, but identical for PA resolution. ‘49’ has two *KIR3DL1*s, while ‘51’ has one.

Three sets of haplotypes were used to constrain genotypic interpretation and estimate frequencies (Fig 2). The names indicate the number of haplotypes in each set: HapSet10, HapSet24, and HapSet63, where HapSet10 ⊂ HapSet24 ⊂ HapSet63 (⊂ denotes ‘a subset of’). The first set, HapSet10 was reported in 2010 by Pyo et al. on 12 unrelated individuals[3]. The latter two sets include haplotypes from family studies by Jiang et al. in 2012[4]; HapSet24 contains all haplotypes with a frequency of 0.1% or greater, and HapSet63 contains all haplotypes described in both reports. The haplotypes are almost exclusively from U.S./U.K. citizens of European descent, although the Pyo cohort contains cB03~tA01, which thus far has only been reported in Africans. Haplotypes were defined according to gene copy number of the 16 KIR genes, as this resolution uniquely identifies all reference structural haplotypes. Since *KIR2DS3* and *KIR2DS5* are paralogous across the centromeric and telomeric regions, they are sometimes informative for haplotype structure based on patterns of linkage disequilibrium; when so, the haplotypes are separated into distinct *KIR2DS3* and *KIR2DS5* versions, while united into one haplotype when not informative. For example, *KIR2DS3* is ubiquitous on cB01~tA01 and therefore treated as one haplotype; however, the presence and location of the two genes in cB01~tA01 is ambiguous and therefore treated as two haplotypes: cB01~tA01_2DS3 and cB01~tA01_2DS5.

### Genotypic interpretation under haplotype constraints

Each individual’s genotype was inferred as potentially-ambiguous pairs of haplotypes (copy number of 16 genes) from one of the reference sets. Inference (see below) was performed using PA resolution for all individuals and again using PA + CNV resolution when available. Some genotypes were interpreted into a pair of haplotypes unambiguously, some ambiguously into multiple pairs, and some genotypes were uninterpretable under the haplotype-pair constraints. When interpretable, haplotype pairs of maximum probability where assigned to each individual.

**Fig 3:**
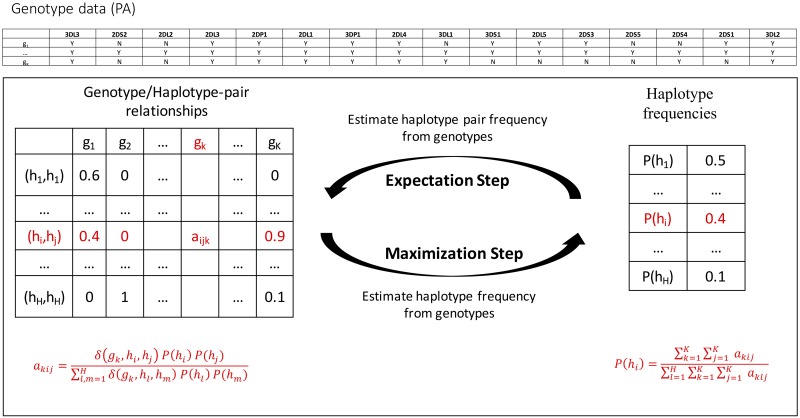
Workflow. Each individual's genotype (g_1_… g_K_) is interpreted as possibly ambiguous pairs of haplotypes (h_1_… h_H_), whose frequencies are initialized evenly. The EM algorithm then alternates between the E and M steps until convergence. The results are haplotype pair assignments for each individual and frequencies estimates for each haplotype.

In Fig 3, the PA genotype data is illustrated in the table ‘Genotype data (PA)’. In the table, each row is a genotype sample and each column is the presence (Y) or absence (N) of a gene in the sample. The table ‘Genotype/Haplotype-pair relationships’ shows how likely a genotype is interpreted by a haplotype-pair. For example, a_ijk_ is the probability that genotype g_k_ is interpreted by the haplotype pair (h_i_,h_j_). a_ijk_ = 0 when (h_i_,h_j_) cannot interpret g_k._

### Haplotype frequency inference

Haplotype frequencies were estimated at PA resolution for all populations (the table ‘Haplotype frequencies’ in Fig 3). Frequencies were also estimated separately at PA + CNV resolution for the half of AFA with CNV data available. Haplotype pairs and cohort frequencies were estimated using an expectation-maximization (EM) algorithm[16] as illustrated in Fig 3. Given a collection of genotypes and a set of reference haplotypes, the EM algorithm assigns to each genotype the haplotype pair with the maximum probability.

Given haplotypes {h_1_, h_2_, … h_H_} and genotypes {g_1_, … g_K_}, the likelihood function is

$$L\left(h_{1},\;h_{2},\;\ldots ,\;h_{H}:g_{1},\;\ldots ,\;g_{K}\right)=\prod_{k=1}^{K}P(g_{k}|h_{1},\;h_{2},\;\ldots ,\;h_{H})=\prod_{k=1}^{K}\prod_{i,j=1}^{H}P{\left(g_{k}|h_{i},\;h_{j}\right)}^{r_{kij}},$$

where r_kij_ is a hidden Bernoulli random variable measuring whether g_k_ is generated from (h_i_,h_j_) and

$$LL\left(h_{1},\;h_{2},\;\ldots ,\;h_{H}:g_{1},\;\ldots ,\;g_{K}\right)=\sum_{k=1}^{K}\sum_{i,j=1}^{H}r_{kij}logP(g_{k}|h_{i},\;h_{j})=\sum_{k=1}^{K}\sum_{i,j=1}^{H}r_{kij}(logP(h_{i})+logP\left(h_{j}\right)),$$

where P(h_i_) is the frequency of haplotype h_i_.

### EM Algorithm

The algorithm iterated between two steps–expectation and maximization—until convergence. After initializing the haplotypes with equal frequency, the expectation step used the current iteration’s estimated haplotype frequencies to calculate haplotype-pair expectations for each genotype, i.e. the entries in the table ‘Genotype/Haplotype-pair relationships’ in Fig 3:

$$a_{kij}=E\left(r_{kij}\right)=P\left(H_{k}=\left(h_{i},\;h_{j}\right)|g_{k}\right)=\frac{\delta \left(g_{k},\;h_{i},\;h_{j}\right)\;P\left(h_{i}\right)\;P\left(h_{j}\right)}{\sum_{l,m=1}^{H}\delta \left(g_{k},\;h_{l},\;h_{m}\right)\;P\left(h_{l}\right)\;P\left(h_{m}\right)}$$

and δ adjusts the statistic for homo/hetero-zygousity:

$$\delta \left(g_{k},\;h_{i},\;h_{j}\right)=1\;if\;h_{i}=h_{j}$$

$$\delta \left(g_{k},\;h_{i},\;h_{j}\right)=2\;if\;h_{i}\neq h_{j}$$

The maximization step used each genotype’s most likely haplotype pair to estimate the frequency of each haplotype illustrated in the table ‘Haplotype frequencies’ in Fig 3:

$$P\left(h_{i}\right)=\frac{\sum_{k=1}^{K}\sum_{j=1}^{K}\;a_{kij}}{\sum_{l=1}^{H}\sum_{k=1}^{K}\sum_{j=1}^{K}\;a_{klj}}$$

An implementation[17] of the algorithm has been deposited in GitHub.

### Statistical analysis

BoxPlotR[18] was used to calculate and plot the distributions.

## Results

### Interpretability of genotypes

**Fig 4:**
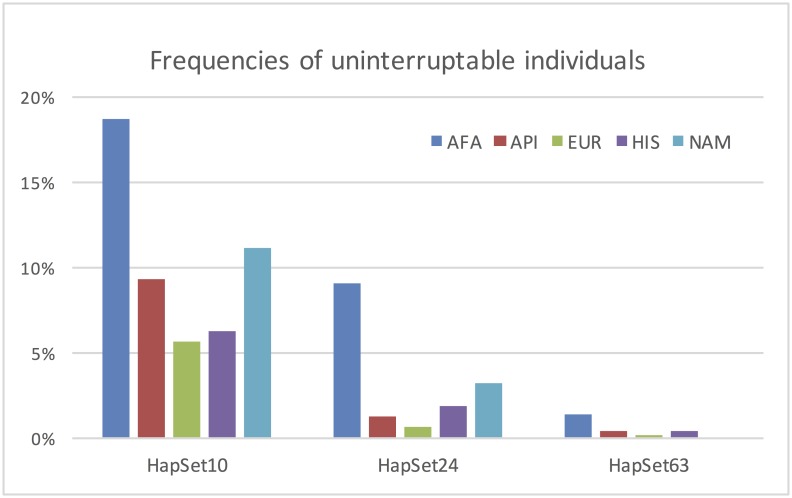
Frequencies of uninterpretable individuals. Each bar represents the percentage of a population that cannot be explained by one of three reference sets of haplotypes. For clarity and distinction, the chart displays a maximum frequency of 20%. Five populations are represented: African American (AFA), Asian Pacific Islander (API), European (EUR), Hispanic (HIS), and Native American (NAM).

Fig 4 plots the fraction of each population that cannot be explained by each of the three sets of reference haplotypes, i.e., the frequency of individuals not covered by the haplotypes under the PA conditions. In the smallest set of haplotypes HapSet10, these frequencies range from 5.6% in Europeans to almost 20% in African Americans. The largest reference set HapSet63 lowers the uninterpretable frequencies to near 0% for all five populations. African Americans exhibit the highest unexplained rates, followed by Native Americans.

**Fig 5:**
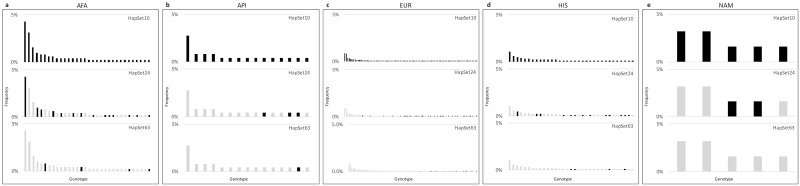
Contributions of individual genotypes to uninterpretable rates. The columns depict the populations and the rows depict the genotype’s fractional contribution of uninterpretable individuals. Black bars mean the haplotype is uninterpretable for the given population and haplotype set, while the grey bars mean the haplotype is interpretable. As bars go from black to grey from row 1 to 3, it indicates the addition of a haplotype (or haplotypes) in the larger set has allowed interpretation of the genotype.

The contribution of each uninterpretable genotype is depicted in Fig 5, which shows the frequency of each population’s PA genotype that cannot be explained by a reference haplotype set. In the plot, the x-axis represents the distinct genotypes and y-axis represents the frequency of the distinct genotypes among the individuals. The black bars indicate that the genotype is uninterpretable by the haplotype set and the grey bars indicate the genotype is interpretable. Almost all the distinct genotypes are near-singletons. If we consider 1–3 occurrence of a genotype as less common, for all the populations, there are approximately 8 of these less common genotypes under HapSet10, and they decrease to 2 under HapSet24, and 1 for HapSet63.

### Estimated PA Haplotype Frequencies

**Fig 6:**
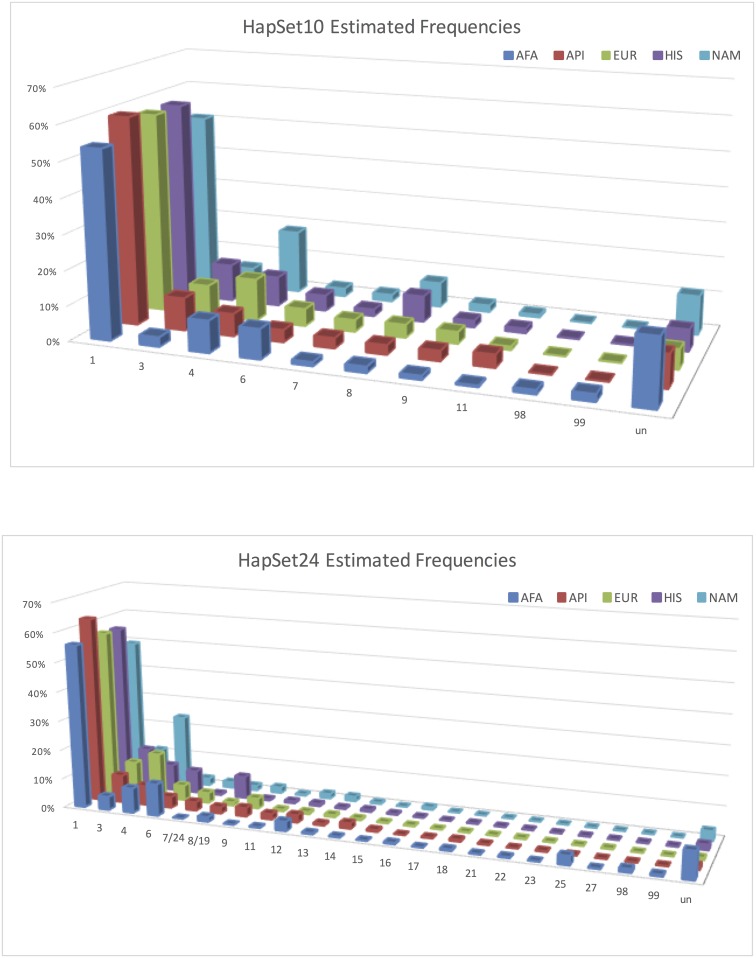
Estimated PA haplotype frequencies for HapSet10 (a) and HapSet24 (b). Frequencies are displayed by population and reference haplotype set. Haplotypes are on the x-axis, frequencies on the y, and populations on the z. For clarity and distinction, the chart displays a maximum frequency of 70%. ‘un’ haplotype represents the frequency of individuals that could not be interpreted.

Fig 6 plots estimated PA haplotypes frequencies (including frequencies of uninterpretable individuals) by population for HapSet10 and HapSet24. Under HapSet10 conditions (Fig 6a), the ratio of uninterpretable individuals are EUR(5.6%), HIS(6.2%), API(9.3%), NAM(11.1%), and AFA(18.7%) with a 13% difference in the range. cA01~tA01 (haplotype 1 in the plot) ranges by 8%, from 51%(NAM) to 59%(API). For API and HIS, the second most common haplotype is cA01~tB01 (haplotype 3 in the plot) (9–11%) and it is cB02~tA01 (haplotype 4 in the plot) (9–18%) for AFA, EUR, and NAM. All other haplotypes have estimated frequencies of less than 10%. cB02~tA01 is the only haplotype with a range of over 10%: 6.4% (API) to 18.26% (NAM).

In the experiment with HapSet24 (Fig 6b), the fourteen additional haplotypes reduce the uninterpretable rates by 4.4% for HIS, 5.0% for EUR, 7.9% for NAM, 8.1% for API, and 9.6% for AFA. Two haplotypes make the biggest contributions: haplotype 25 in AFA (2.9%) and haplotype 12 in AFA (3.1%), API (2.5%), and NAM (1.6%). Haplotype 25 is similar to the more common cB01~tA01 except the absence of 2DS3. Haplotype 12 (cB04~tB03) is a deleted form of cB01~tB01 such that the *KIR2DL5* in the centromeric region becomes proximal to the *KIR2DS3S5* in the telomeric region. The frequencies of the original ten haplotypes do not appreciably change except in NAM, where three haplotypes had altered frequencies of 5.5%: the cB02 previously associated with tB01 was instead associated with tA01 and cA01~tB01 was more frequent.

**Fig 7:**
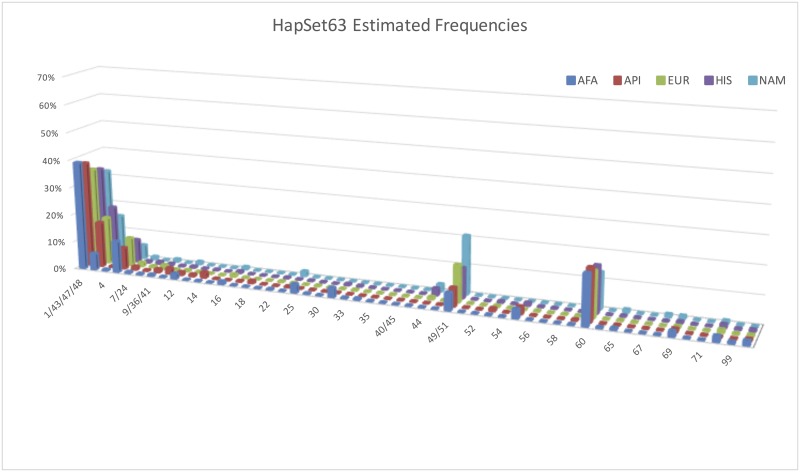
Estimated PA haplotype frequencies for HapSet63. Note the spikes for haplotypes 49/51 and 60. Frequencies are displayed by population and reference haplotype set. Haplotypes are on the x axis, frequencies on the y, and populations on the z. For clarity and distinction, the chart displays a maximum frequency of 70%. ‘un’ haplotype represents the frequency of individuals that could not be interpreted. Combined haplotypes (e.g. 7/24) indicate CNV ambiguity under PA conditions (see Methods).

In the experiment with HapSet63 (Fig 7), the forty-three additional haplotypes further lower the uninterpretable rates by 0.4% for EUR, 0.8% for API, 1.4% for HIS, 3.2% for NAM, and 7.7% for AFA. The maximum uninterpretable rate is now 1.4% for AFA and < = 0.4% for the other populations. The biggest new contributions are dramatic in two haplotypes, ‘49/51’ (6–21%) and ‘60’ (13–18%) in all populations. Haplotype ‘49/51’ is cB02~tA01 with *KIR2DP1~KIR2DL1*. Haplotype ‘60’ is cA01~tA01 without *KIR3DL3*. S1 Table contains all frequencies.

### Estimated CNV Haplotype Frequencies

**Fig 8:**
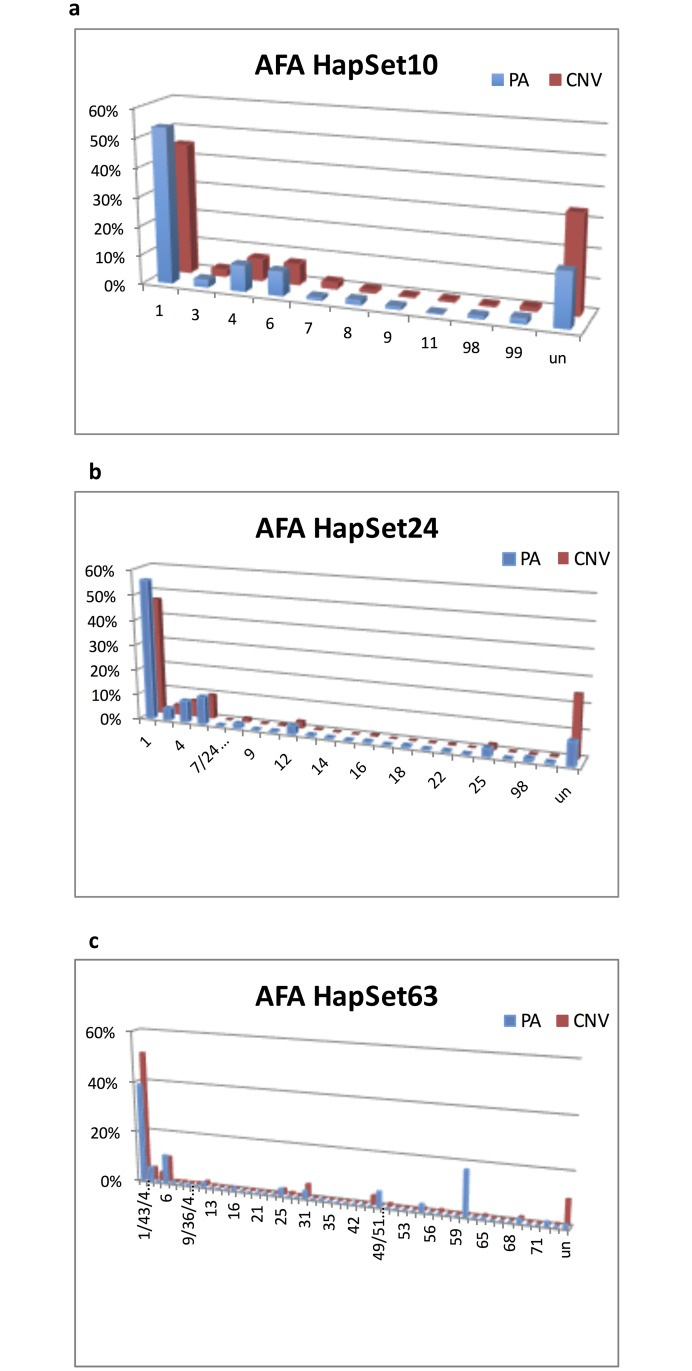
Estimated PA + CNV haplotype frequencies for AFA. Frequencies are plotted by typing resolution and reference haplotype set. Haplotypes are on the x-axis, frequencies on the y, and populations on the z. Each PA haplotype may be equivalent to multiple CNV haplotypes; for example, CNV haplotypes 7 and 24 are equivalent at PA resolution.

Haplotype frequencies were additionally estimated using the CNV data available in 52% of the individuals in the AFA population. The results are plotted in Fig 8, along with the equivalent PA estimates for comparison.

Comparing the estimates by PA to CNV resolutions in the experiment with HapSet10 (Fig 8a), haplotype 1 (cA01~tA01) falls 8% from 53% to 45%. Meanwhile the uninterpretable rate rises 14% from 19% to 33%. The estimates for the other haplotypes are fairly consistent between the resolutions. The patterns are very similar in the experiment with HapSet24 (Fig 8b). When increasing the resolution from PA to CNV, cA01~tA01 falls 9% from 56% to 47%. Meanwhile, the uninterpretable rate rises 15% from 9% to 24%. Again only slight differences are seen for the other haplotypes. The patterns are more complicated in the experiment with HapSet63 (Fig 7c). The uninterpretable rate similarly rises 9%, from 1% to 10%. However, the frequency of cA01~tA01 rises 12% from 39% to 51% instead of falling. The two new haplotypes with the highest frequency, ‘49/51’ (6%) and ‘60’ (18%), fell to less than 3% and 1% respectively.

**Fig 9:**
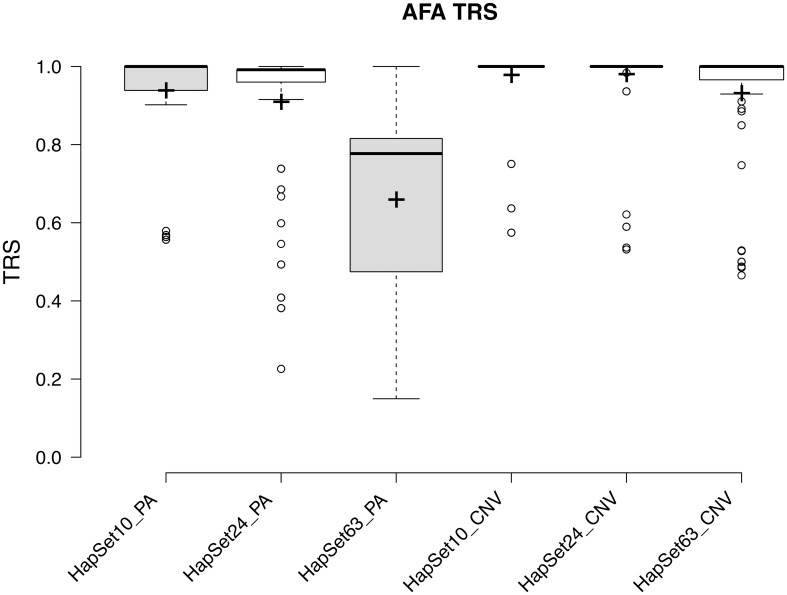
TRS distribution under PA and CNV resolutions for each HapSet. Typing resolution scores are plotted for every AFA for whom CNV typing was available. Center lines show the medians; box limits indicate the 25th and 75th percentiles as determined by R software; whiskers extend 1.5 times the interquartile range from the 25th and 75th percentiles. All data points beyond this are outliers represented by dots. Crosses represent sample means; width of the boxes is proportional to the square root of the sample size. The size of each sample is labeled in the x-axis.

Fig 9 quantifies the ambiguity of PA resolution relative to CNV as distribution of typing resolution scores (TRS)[19]:

$$TRS=\sum p^{2}$$

where *p* is a set of normalized haplotype frequencies. It is bounded on [0, 1] interval, where 0 indicates maximum ambiguity, and 1 indicates no ambiguity. As such, TRS can be used to compare typings generated across different systems.

The three leftmost distributions plot TRS using PA resolution for the three reference haplotype sets. The rightmost three plot them for CNV resolution. When resolution is fixed, larger haplotype sets provide lower averages, higher variances, and larger sample sizes. When haplotype sets are fixed, higher resolution provides higher averages, lower variances, and lower sample sizes. The most dramatic difference between the two resolutions occurs when HapSet63 is used: the median TRS (as indicated bold horizontal bars) rises from 0.78 to 1.00, and the first quartile rises from 0.47 to 0.97. That is, under CNV resolution, outliers explain all but 3% of the ambiguity.

## Discussion

The primary purposes of this study were to estimate KIR haplotype frequencies in a relatively large cohort, and to evaluate the variations of the frequencies by population, reference haplotypes, and typing resolution. Modern KIR association studies can improve genotype-only analysis by increasing resolution to reduce ambiguity and include cofactors to improve functional interpretations. For example, some commonly analyzed co-factors include MHC and peptide binding partners, expression levels, and NK-repertoire. The fact that several haplotypic effects have been published despite the relative lack of haplotype resolution data, may suggest a larger role for haplotype marker analysis in future studies, either as a single factor or in cooperation with others.

### Interpretability of genotypes

Larger sets of reference haplotypes decrease the frequency of unexplainable individuals, as shown in Figs 4 and 5. For all populations except AFA, the additional haplotypes from HapSet10 to HapSet24 represent the largest changes in frequencies, and result in uninterpretable frequencies near zero. AFAs, on the other hand, see a relatively large change in HapSet24 and even larger with the additional haplotypes in HapSet63. Even so, 10% are still not explainable under CNV consideration. The large change with HapSet63 demonstrates the haplotypic diversity of AFAs and how some relatively rare EUR haplotypes may explain some relatively common haplotypes in AFAs.

Four non-singleton uninterpretable genotypes resolved in HapSet24 are mainly due to the additions in AFA, API, and NAM of cB04~tB03 (with *KIR2DS3S5*) and cB03~tA01 (with *KIR2DS5*). Three other non-singleton uninterpretable genotypes resolved in HapSet63 in the EUR and HIS groups are more diverse and could not obviously be resolved by the addition of a specific haplotype or two. HapSet63 provides parsimonious coverage for all populations and both typing resolutions conditions.

### Estimated Haplotype Frequencies

We compared the haplotype frequency estimates for HapSet10 with three European-based [3][4][15] and one Native American study[20] (S2 Table). All estimates were consistent relative to the previous studies to within 5% except for two haplotypes in the NAM population. Our results showed cB02 associated more frequently with tA01 (+14%) than tB01 (-8.5%). The difference is even stronger when CNV resolution is considered. There are several possible explanations for this difference, including pathogen selection. However, our evidence may support founder effects combined with genetic drift as previously speculated [21], since the comparison is between U.S. based NAMs relative to various native populations from south of the U.S. On the other hand, the differences could be due to the potentially mixed nature of either group. Since no further breakdown was available for our 63 individuals, we aggregated the southern group for comparison. The haplotype that exhibits the widest range of frequencies across populations is cB02~tA01, which contains a centromeric B haplotype lacking the *KIR2DL5*~*KIR2DS3S5*~*KIR2DL1* region. Comparisons for the three other minority populations are not possible at this time due to our admixed and otherwise biased U.S.-based registry.

The distinguishing features of our computational approach relative to other[22][23][24][25][26][27][20][15][28], also mainly EM approaches, are the ability to use multiple or mixed resolutions as well as the size of data sets to which we have applied it.

### Population

The distribution of the five populations roughly reflects those of the U.S. in general and the Be The Match Registry^®^ that serves it. Ideally, genotypes would be interpreted in the context of population-specific haplotypes. These sets should be available soon, with the adoption of longer-range sequencing and continued research in minority groups. On the other hand, HapSet63 likely is not missing any common population-specific haplotypes since explains all genotypes fairly well except for rare/uncommon genotypes (at least parsimoniously). Since identical genotypes in multiple populations may have derived from different haplotype pairs, genotypes should be resolved with population specific haplotypes.

### Resolution

It can be misleading to interpret PA data with a reference haplotype set that is too large, as shown for the largest set in Fig 8. Frequencies of uninterpretable genotypes and cA01~tA01 are much lower compared with the smaller HapSet24. Two of the 39 new haplotypes included in HapSet63 have estimated frequencies >10%. These estimates are shown to be erroneous under CNV conditions. The first haplotype, 60, is haplotype 1 (cA01~tA01) without *KIR3DL3*. The estimated frequency (18%) is much too high for a gene generally reported to be absent less than 1% of the time[4] and is estimated at 0.6% in our CNV cohort. This is at the expense of haplotype 1’s frequency, which is estimated at 39% instead of 51% for CNV. The second haplotype, 49/51, is haplotype 4 (cB02~tA01) with *KIR2DP1~KIR2DL1*. Its PA estimated frequency is 6%, compared with 0% for CNV. This is at the expense of haplotype 4’s frequency, which is estimated at 0.03% instead of 4% for CNV.

These inflated frequencies illustrate two larger issues that can occur when interpreting low resolution in the context of a large reference haplotype set. Both issues involve the situation when a haplotype is a gene content subset of the other. From the point of view of PA interpretation, one haplotype can be ‘hidden’ behind another and result in misleading estimates.

The first situation can occur when the superset haplotype is relatively frequent. It can lead to the subset haplotype ‘stealing’ frequency from the superset haplotype. For example, haplotype 60 is a subset of haplotype 1: it is a cA01~tA01 haplotype without *KIR3DL3*. Except for the ubiquity of *KIR3DL3*, the PA data provides no distinction between 60 and 1 and therefore leads to a higher than expected frequency for 60.

The second situation is the opposite of the first: it can occur when a reference set contains a haplotype that is a gene content superset of a less frequent haplotype. It can lead to the superset haplotype ‘stealing’ frequency from the subset because adding the relatively frequent gene(s) expands the number of genotypes that can be explained. For example, haplotype 49/51 is a superset of haplotype 4: it is a cB02~tA01 haplotype with *KIR2DP1*~*KIR2DL1*. Both of those genes have high frequencies such that adding them makes it more accommodating for genotypes with those two usually common genes. This leads to misestimates of haplotype 49/51 with a frequency of 6% and haplotype 4 dropping from 8% to 0.3%. From our CNV analysis, haplotype 4 is exclusively inherited with haplotype 1, and haplotype 49/51 is exclusively inherited with haplotype 16.

Further experiments with mixed PA and CNV resolution show these two situations cannot be remedied by mixed case genotyping wherein some genes (e.g., the four framework genes) are typed at CNV resolution and the rest at PA. In summary, we suggest that reference haplotypes for PA data should not contain haplotypes that are gene-content subsets of other haplotypes. Such a situation can cause hidden haplotypes to erroneously unbalance its frequency with its ‘non-hidden’ partner: rare hidden haplotypes can be inflated and common hidden haplotypes can be the deflated. This consideration can be valuable for retrospective studies by informing the selection of reference haplotypes given typing resolution(s). It can also be valuable for prospective studies by suggesting which genes should be typed at certain resolutions given a reference set.

The substantial increased clarity that CNV provides is clearly shown when comparing the ambiguity distributions for PA and CNV in Fig 9. Higher resolution increased clarity for every reference set. Using the largest set, the higher resolution provided unambiguous haplotype-pair assignments for the majority of individuals, resulting in a 22% higher median TRS.

In summary, we generated haplotype frequency estimates for over 10,000 individuals in five populations, we reported on the effects of reference haplotype set and resolution, and we documented the inaccuracies that may occur when using low resolution and large sets of reference haplotypes. These analytical methods and novel predictions may allow for finer-grained KIR-association analysis, especially when combined with other factors such as expression and HLA ligands.

## Supporting Information

S1 Table Estimated PA haplotype frequencies by population and reference haplotype set. Haplotypes are on the x-axis, frequencies on the y, and populations on the z. For clarity and distinction, the chart displays a maximum frequency of 70%. ‘un’ haplotype represents the frequency of individuals that could not be interpreted.
(DOCX)

S2 Table Frequency comparisons with previous studies. HapSet10 frequency estimates compared with a) two EUR studies and b) one NAM study.
(DOCX)
