# Supplementary material for: Estimating KIR Haplotype Frequencies on a Cohort of 10,000 Individuals: A Comprehensive Study on Population Variations, Typing Resolutions, and Reference Haplotypes
Source: PLoS One. 2016 Oct 10;11(10):e0163973. doi: 10.1371/journal.pone.0163973 (PMC5056762; doi:10.1371/journal.pone.0163973)
Supplement: S1 Table — Haplotypes are on the x-axis, frequencies on the y, and populations on the z. For clarity and distinction, the chart displays a maximum frequency of 70%. ‘un’ haplotype represents the frequency of individuals that could not be interpreted. (DOCX) [file pone.0163973.s001.docx]

**Supplemental Table 1.** Estimated PA haplotype frequencies by population and reference haplotype set.

| **HapSet10 Frequencies** | | | | | |
| --- | --- | --- | --- | --- | --- |
| Haplotype | AFA | API | EUR | HIS | NAM |
| 1 | 53.49 | 59.29 | 57.23 | 57.23 | 50.80 |
| 3 | 2.71 | 9.30 | 8.64 | 10.62 | 5.56 |
| 4 | 9.07 | 6.39 | 12.01 | 8.48 | 18.26 |
| 6 | 8.49 | 3.49 | 4.96 | 4.52 | 2.38 |
| 7 | 1.11 | 2.91 | 3.40 | 1.96 | 1.98 |
| 8 | 1.74 | 2.71 | 3.74 | 7.49 | 7.15 |
| 9 | 1.11 | 2.91 | 3.40 | 1.96 | 1.98 |
| 11 | 0.39 | 3.68 | 0.99 | 1.35 | 0.79 |
| 98 | 1.16 | 0.00 | 0.01 | 0.09 | 0.00 |
| 99 | 2.02 | 0.00 | 0.04 | 0.09 | 0.00 |
| Uninterpretable | 18.70 | 9.30 | 5.60 | 6.20 | 11.10 |
| Total | 100.00 | 100.00 | 100.00 | 100.00 | 100.00 |

| **HapSet24 Frequencies** | | | | | |
| --- | --- | --- | --- | --- | --- |
| Haplotype | AFA | API | EUR | HIS | NAM |
| 1 | 55.68 | 62.76 | 56.20 | 55.95 | 49.19 |
| 3 | 4.54 | 9.30 | 11.38 | 13.65 | 11.11 |
| 4 | 8.40 | 6.59 | 15.26 | 8.81 | 23.80 |
| 6 | 10.81 | 3.68 | 5.03 | 7.59 | 2.38 |
| 7/24 | 0.00 | 2.90 | 3.40 | 0.44 | 1.98 |
| 8/19 | 1.55 | 2.13 | 1.02 | 7.45 | 1.59 |
| 9 | 0.00 | 2.90 | 3.40 | 0.44 | 1.98 |
| 11 | 0.19 | 1.84 | 0.50 | 0.68 | 0.40 |
| 12 | 3.18 | 2.52 | 0.74 | 0.80 | 1.59 |
| 13 | 0.38 | 0.58 | 0.70 | 0.42 | 1.59 |
| 14 | 0.19 | 1.84 | 0.50 | 0.68 | 0.40 |
| 15 | 0.00 | 0.58 | 0.20 | 0.32 | 0.00 |
| 16 | 0.38 | 0.00 | 0.23 | 0.19 | 0.79 |
| 17 | 0.00 | 0.20 | 0.06 | 0.00 | 0.00 |
| 18 | 0.48 | 0.58 | 0.11 | 0.14 | 0.00 |
| 21 | 0.10 | 0.20 | 0.11 | 0.09 | 0.00 |
| 22 | 0.29 | 0.00 | 0.23 | 0.14 | 0.00 |
| 23 | 0.00 | 0.20 | 0.05 | 0.05 | 0.00 |
| 25 | 2.99 | 0.00 | 0.10 | 0.14 | 0.00 |
| 27 | 0.10 | 0.00 | 0.15 | 0.05 | 0.00 |
| 98 | 1.15 | 0.00 | 0.02 | 0.09 | 0.00 |
| 99 | 0.48 | 0.00 | 0.03 | 0.09 | 0.00 |
| Uninterpretable | 9.10 | 1.20 | 0.60 | 1.80 | 3.20 |
| Total | 100.00 | 100.00 | 100.00 | 100.00 | 100.00 |

| **HapSet63 Frequencies** | | | | | |
| --- | --- | --- | --- | --- | --- |
| Haplotype | AFA | API | EUR | HIS | NAM |
| 1/43/47/48 | 38.97 | 37.80 | 34.46 | 33.55 | 31.76 |
| 3/28/37/38 | 5.79 | 16.09 | 16.71 | 19.53 | 15.08 |
| 4 | 0.29 | 0.19 | 2.38 | 2.05 | 4.76 |
| 6 | 11.19 | 7.56 | 9.97 | 8.06 | 4.76 |
| 7/24 | 0.38 | 1.07 | 1.23 | 0.47 | 0.79 |
| 8/19 | 0.20 | 0.19 | 0.36 | 0.70 | 0.00 |
| 9/36/41 | 0.38 | 1.07 | 1.23 | 0.47 | 0.79 |
| 11 | 0.05 | 1.84 | 0.09 | 0.16 | 0.00 |
| 12 | 1.74 | 0.97 | 0.34 | 0.37 | 0.79 |
| 13 | 0.00 | 0.58 | 0.20 | 0.09 | 0.00 |
| 14 | 0.05 | 1.84 | 0.09 | 0.16 | 0.00 |
| 15 | 0.00 | 0.00 | 0.04 | 0.00 | 0.00 |
| 16 | 0.87 | 0.00 | 0.70 | 0.51 | 0.79 |
| 17 | 0.00 | 0.19 | 0.06 | 0.00 | 0.00 |
| 18 | 0.00 | 0.78 | 0.01 | 0.00 | 0.00 |
| 21 | 0.00 | 0.00 | 0.00 | 0.00 | 0.00 |
| 22 | 0.15 | 0.00 | 0.16 | 0.19 | 0.00 |
| 23 | 0.00 | 0.19 | 0.05 | 0.05 | 1.59 |
| 25 | 2.90 | 0.00 | 0.00 | 0.09 | 0.00 |
| 27 | 0.10 | 0.00 | 0.14 | 0.05 | 0.00 |
| 30 | 0.00 | 0.00 | 0.00 | 0.00 | 0.00 |
| 31 | 3.09 | 0.00 | 0.00 | 0.37 | 0.00 |
| 33 | 0.15 | 0.00 | 0.16 | 0.19 | 0.00 |
| 34 | 0.38 | 0.00 | 0.01 | 0.05 | 0.00 |
| 35 | 0.00 | 0.00 | 0.01 | 0.00 | 0.00 |
| 39 | 0.00 | 0.00 | 0.03 | 0.00 | 0.00 |
| 40/45 | 0.00 | 0.00 | 0.00 | 0.00 | 0.00 |
| 42 | 0.00 | 0.00 | 0.01 | 0.00 | 0.00 |
| 44 | 0.00 | 0.00 | 0.69 | 2.28 | 2.38 |
| 46 | 0.00 | 0.00 | 0.01 | 0.00 | 0.00 |
| 49/51 | 6.07 | 6.40 | 13.00 | 10.58 | 20.64 |
| 50 | 0.00 | 0.00 | 0.00 | 0.00 | 0.00 |
| 52 | 0.00 | 0.00 | 0.00 | 0.00 | 0.00 |
| 53 | 0.10 | 0.78 | 0.23 | 0.37 | 0.00 |
| 54 | 0.00 | 0.00 | 0.00 | 0.00 | 0.00 |
| 55 | 3.09 | 2.52 | 0.02 | 0.84 | 0.00 |
| 56 | 0.00 | 0.00 | 0.07 | 0.09 | 0.00 |
| 57 | 0.00 | 0.00 | 0.00 | 0.09 | 0.00 |
| 58 | 0.00 | 0.00 | 0.00 | 0.00 | 0.00 |
| 59 | 0.10 | 0.19 | 0.03 | 0.00 | 0.00 |
| 60 | 17.84 | 18.41 | 15.92 | 16.40 | 12.70 |
| 62 | 0.20 | 0.00 | 0.00 | 0.00 | 0.00 |
| 65 | 0.48 | 0.00 | 0.10 | 0.14 | 0.79 |
| 66 | 0.00 | 0.00 | 0.05 | 0.05 | 0.00 |
| 67 | 0.00 | 0.00 | 0.00 | 0.00 | 0.00 |
| 68 | 0.00 | 0.00 | 0.00 | 0.05 | 0.79 |
| 69 | 1.74 | 0.97 | 0.34 | 0.37 | 0.79 |
| 70 | 0.00 | 0.00 | 0.02 | 0.00 | 0.00 |
| 71 | 0.00 | 0.00 | 0.00 | 0.00 | 0.00 |
| 98 | 1.83 | 0.00 | 0.83 | 1.12 | 0.79 |
| 99 | 0.48 | 0.00 | 0.07 | 0.14 | 0.00 |
| Uninterpretable | 1.40 | 0.40 | 0.20 | 0.40 | 0.00 |
| Total | 100.00 | 100.00 | 100.00 | 100.00 | 100.00 |
