# Supplementary material for: Estimating KIR Haplotype Frequencies on a Cohort of 10,000 Individuals: A Comprehensive Study on Population Variations, Typing Resolutions, and Reference Haplotypes
Source: PLoS One. 2016 Oct 10;11(10):e0163973. doi: 10.1371/journal.pone.0163973 (PMC5056762; doi:10.1371/journal.pone.0163973)
Supplement: S2 Table — HapSet10 frequency estimates compared with a) two EUR studies and b) one NAM study. (DOCX) [file pone.0163973.s002.docx]

Supplemental Table 2. Frequency comparisons with previous studies. HapSet10 frequency estimates compared with a) two EUR studies and b) one NAM study.

a.

b.
